# Case epidemiology from the first three years of a pilot laboratory-based surveillance system for elevated blood-lead concentrations among children in England, 2014–17: implications for public health action

**DOI:** 10.1093/pubmed/fdz024

**Published:** 2019-05-24

**Authors:** D J Roberts, Helen Crabbe, Tayo Owodunni, Harriet Gordon-Brown, Rebecca Close, Shanel Reshat, Barry Sampson, Ruth Ruggles, Gavin Dabrera, Araceli Busby, Giovanni Leonardi

**Affiliations:** 1 Field Epidemiology Training Programme Public Health England, Colindale, London, UK; 2 European Programme for Intervention Epidemiology Training (EPIET), European Centre for Disease Prevention and Control, (ECDC), Stockholm, Sweden; 3 Environmental Epidemiology Group, Centre for Radiation, Chemical and Environmental Hazards, Public Health England, Chilton, UK; 4 North East & North Central London Health Protection Team, Public Health England, London, UK; 5 London Imperial Charing Cross Hospital Supra-regional Assay Service Trace Elements Laboratory, Charing Cross Hospital, London, UK; 6 Public Health England, Colindale, London, UK; 7 National Infection Service, Public Health England, Colindale, London, UK

**Keywords:** children, environment, epidemiology

## Abstract

**Background:**

Children incur lead toxicity even at low blood-lead concentrations (BLCs), and testing in England is opportunistic. We described epidemiology of cases notified to a passive laboratory-based surveillance system (SS), the Lead Poisoning in Children (LPIC) SS to inform opportunities to prevent lead exposure in children in England.

**Methods:**

Surveillance population: children <16 years of age and resident in England during the reporting period September 2014–17. Case definition: children with BLC ≥0.48 μmol/l (10 μg/dl). We extracted case demographic/location data and linked it with laboratory, area-level population and socio-economic status (SES) data. We described case BLCs and calculated age-, gender- and SES-specific notification rates, and age-sex standardised regional notification rates.

**Results:**

Between 2014 and 2017 there were 86 newly notified cases, giving an annual average notification rate of 2.76 per million children aged 0–15 years. Regionally, rates varied from 0.36 to 9.89 per million. Rates were highest in the most deprived quintile (5.38 per million), males (3.75 per million) and children aged 1–4 years (5.89 per million).

**Conclusions:**

Males, children aged 1–4 years, and children in deprived areas may be at higher risk, and could be targeted for primary prevention. Varied regional notification rates suggest differences in clinician awareness of lead exposure and risk factors; guidelines standardising the indications for BLC-testing may assist secondary prevention.

## Background

Exposure to lead can result in severe multi-system toxicity. Blood-lead concentration (BLC) is measured to determine recent exposure, as the half-life of lead in blood is ~30 days.^1^ Overt manifestations of toxicity (i.e. lead poisoning), such as anaemia or abdominal pain, accompany higher lead concentrations, e.g. BLCs >1.93 μmol/l (40 μg/dl)^2^ (note this BLC is not intended to give a definitive cut-off at which frank symptoms will appear). Lead exposures resulting in a lower BLC may present sub-clinically, but still cause harm: decreased intellectual function and other neuro-behavioural problems are associated with BLCs even below 0.48 μmol/l (10 μg/dl), with no lower threshold for toxicity.^2–4^

BLCs in children have fallen dramatically in high-income settings.^5,6^ However, lead is a persistent contaminant, therefore current exposures often reflect historic use. In high-income countries, ingestion is now the primary route of exposure, particularly from flakes and dust from exposed leaded paint,^3,5^ which was widely used in domestic settings prior to its gradual withdrawal (from the 1960s in the UK^7^). Children can also be exposed to lead in food, soil or water, consumer goods or medicines (including traditional and complementary preparations) that do not meet regulatory standards for lead content,^2^ and indirectly due to their guardian’s occupation.^8^ Lead exposure is more common in younger children,^6^ and children with pica^9^ or developmental delay,^10^ an association probably mediated by increased hand to mouth behaviour, with which lead exposure also correlates.^11^ Lead exposure may also contribute to developmental delay.^12^ Measurement of BLC is therefore recommended in children presenting with pica or developmental delay potentially exposed to ingestible lead hazards.^10,13,14^ There are no recent estimates of population-level exposure to lead in children in England, but a 2008–09 survey from France estimated 0.09% of children aged 1–6 years had a BLC ≥0.48 μmol/l (10 μg/dl) and 1.5% a BLC ≥0.24 μmol/l (5 μg/dl).^11^ Population lead exposure is strongly influenced by setting, but these findings may be broadly similar to the situation in England.

Case detection depends on clinician awareness of risk factors for exposure and presenting symptoms and signs, as evidence was considered insufficient to support population screening in the UK.^15^ Notification to Public Health England (PHE) Health Protection Teams (HPTs), though not a statutory requirement, is encouraged if the BLC meets the ‘public health action level’ of ≥0.48 μmol/l (10 μg/dl).^16^ Case-management includes steps to systematically identify and remove the potential source(s) of lead exposure, and consider if other people, particularly household members, have also been exposed.^17^

The surveillance of elevated blood-lead in children (SLiC) in the UK and Ireland study developed case reporting pathways for lead exposure, and recommended implementation of a passive, laboratory-based pilot surveillance system (SS).^18^ The lead poisoning in children (LPIC) SS was implemented in March 2014 in England with the aim to increase case reporting to PHE, and to decrease the time between case diagnosis and initiation of public health action to prevent further exposure to lead hazards.^19^ Evaluation in 2016 showed LPIC had met these case-level management aims, and recommended permanent implementation,^20^ and it is now known as the Lead Exposure in Children Surveillance System (LEICSS).^21^ Without screening or recent survey data, surveillance of cases identified by clinicians offers a means of gathering intelligence to guide public health action to prevent further cases. SLiC provided some information about cases, but only accrued a small number of notifications.^18^ We therefore aimed to describe LPIC-case epidemiology, to inform opportunities for primary and secondary prevention of lead exposure in children in England.

## Methods

### Surveillance system overview

**Fig. 1:**
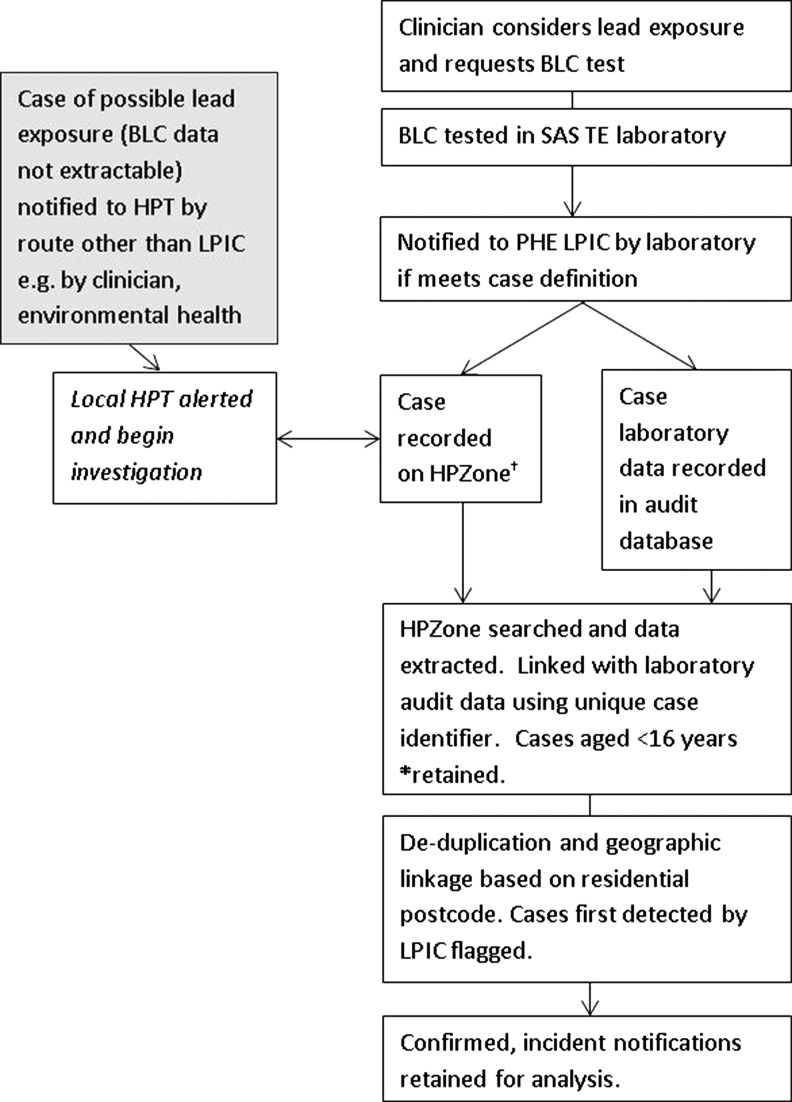
Flow diagram illustrating case notification to the LPIC surveillance system, and other routes of notification to PHE HPTs (grey box) and data selection and management for analysis, England 1 September 2014–31 August 2017. BLC, blood-lead concentration; SAS, Supra-regional assay service; TE, Trace Elements; PHE, Public Health England; LPIC, lead poisoning in children; HPT, health protection team. †HPZone is the public health case-management system in England. *At date of specimen, or date of entry onto HPZone if specimen date missing.

Figure 1 summarises the process of case notification to LPIC as well as data management for this analysis. Cases were notified by Supra-Regional Assay Service (SAS) Trace Elements laboratories, of which there were six such laboratories in England.^22^ PHE was also notified of possible cases by sources other than SAS laboratories, for example by clinicians, who may have notified local HPTs directly. These cases would also have been entered onto HPZone (the public health case-management system in England) by the investigating HPT, but BLC may have been <0.48 μmol/l (10 μg/dl), and BLC data recording was not structured to permit data extraction, hence we did not include these cases in our main analysis.

### Surveillance population

Children aged <16 years and resident in England during the analysis period 01 September 2014–31 August 2017.

### Case definition

A child with a BLC ≥0.48 μmol/l (10 μg/dl).

### Case data recording

LPIC surveillance officers entered case details onto the HPZone system following a standard operating procedure, prior to case report to the relevant local HPT. Laboratory data (initial BLC and specimen date at notification only) were entered onto an Excel database for audit purposes.

### Case identification and selection for this study

We searched HPZone across all PHE HPTs on 26 October 2017 for cases notified from 01 January 2012 with ‘lead’ as the ‘agent of exposure’. We selected cases with a specimen date (or date of entry onto HPZone if their specimen date was missing) from 01 September 2014–31 August 2017. We did not use the first 6 months of data from LPIC initiation to allow a system implementation period.

### Data extraction and management

We extracted case administrative, vital status, demographic and postcode data and linked these using the HPZone unique case identifier with LPIC laboratory data. We excluded cases aged 16 years or older on the specimen date (or date of entry to HPZone if specimen date was missing), and we also checked residential postcodes were based in England. We de-duplicated cases using HPZone number and NHS number, or surname-postcode combination if the NHS number was missing. We flagged cases as first entered onto HPZone by LPIC SS officers (and therefore first notified by SAS laboratories) using HPZone case administrative data. We linked records to area-level population (2015 Office for National Statistics mid-year population estimates for 0–15-year-olds for PHE regions) and 2015 decile of index of multiple deprivation (IMD; using Lower layer Super Output Area 2011 boundaries) using case postcode of residence at notification. We excluded cases notified to HPTs by sources other than the LPIC participating laboratories because we were unable to confirm the BLC for these cases. However, we retained cases first notified to HPTs for which LPIC had also subsequently received a notification from one of the six laboratories participating in surveillance, provided the specimen date relating to this notification was the same or earlier than the date of first report to the local HPT. We defined household by cases who shared the same postcode and surname.

### Data analysis

We described case counts and characteristics, and calculated the average annual case notification rate between 01 September 2014 and 31 August 2017 by age group (<1 year, 1–4 years, 5–11 years and 12–15 years), gender, quintile of IMD and PHE-region. We calculated the mean difference and range in age between consecutive cases (by specimen date) for household clustered cases. For rates, we took the period case count as the numerator and the PHE-region population estimate multiplied by three as the denominator. We calculated 2014–17 regional directly standardised rates (DSR) standardised for age and gender using England 2015 mid-year population estimate. We calculated relative DSRs by dividing the regional DSR by the DSR for the region with the lowest rate. We described the distribution of case BLCs, and because of the non-normal distribution, used the Kruskal–Wallis test to statistically test for a difference in BLCs by age group, and by gender. We repeated the description of case characteristics and calculation of crude national and regional rates as a sensitivity analysis, this time including all children <16 years old recorded on HPZone with lead as the agent of exposure, to consider the impact of including cases not reported to LPIC.

All analyses were performed in STATAv14 (StataCorp, USA), and the manuscript written to adhere to ‘STrengthening the Reporting of OBservational studies in Epidemiology’ reporting guidelines. PHE, on behalf of the Secretary of State for Health and Social Care, has the authority under Section 251 of the NHS Act 2006 to process and analyse confidential data for the purpose of disease surveillance and control, therefore no other ethical permissions were sought for this study.

## Results

### Cases reported

**Fig. 2:**
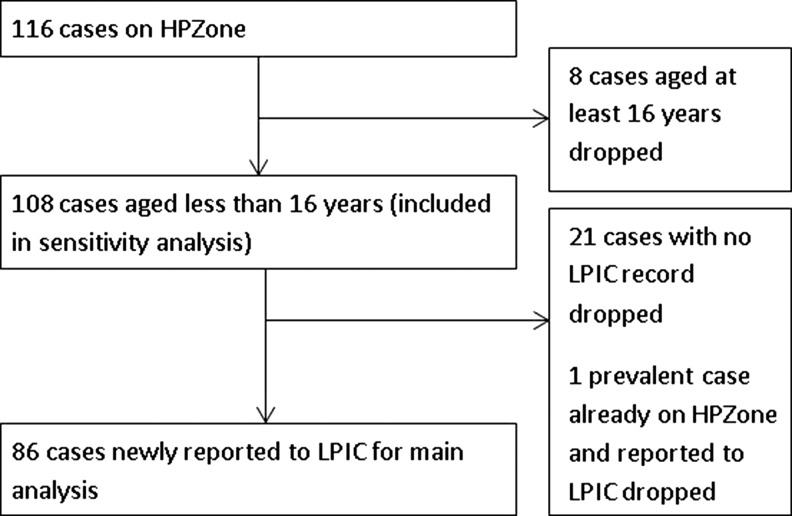
Cases of exposure to lead on HPZone, and LPIC cases retained for analysis, England, 1 September 2014–31 August 2017.

The number of cases included/excluded in this study is shown in Fig. 2. Three of 86 incident cases did not have a specimen date. The number of cases notified to LPIC increased each year, from 23/86 (27%) in 2014–15, 26/86 (30%) in 2015–16, to 37/86 (43%) in 2016–17. About 10/86 (12%) cases shared a postcode and surname (two cases each in five households). The mean difference in age between consecutive clustered cases in each household was −0.2 years (range −6 to +7 years, two were the same age). The average annual notification rate for 2014–17 was 2.76 per million children aged 0–15 years. Four of the total 86 cases were first reported directly to a HPT, meaning 82/108 (76%) cases aged <16 years old notified to PHE over the period were first reported via LPIC.

### Case characteristics

Table 1 lists the case numbers and notification rates by characteristic. Median age was 4 years (interquartile range, IQR 2–7 years). Rates generally increased with increasing deprivation, were higher in males compared with females, and highest in 1–4 year olds compared with other age groups. The median BLC in 86 cases was 0.78 μmol/l (IQR 0.55–1.09; 16.10 μg/dl (IQR 11.39–22.6)), mean 1.13 μmol/l (23.39 μg/dl). BLC was <1.93 μmol/l (40 μg/dl) in 80/86 (93%) cases. One case was deceased, and the BLC in this case was 17.59 μmol/l (364 μg/dl), the highest observed. There was no strong statistical evidence BLC varied by age group (*P* = 0.431) or gender (*P* = 0.129).

**Table 1:** Count and percentage of cases of cases of exposure to lead, and notification rates by descriptive characteristic, England, 1 September 2014–31 August 2017 (*n* = 86)

Characteristic	Sub-group	Count of cases	Percent	Rate per million
Index of multiple deprivation of residence	1—Least deprived	6	7	1.05
	2	6	7	1.07
	3	13	15	2.24
	4	20	23	3.09
	5—Most deprived	41	48	5.38
Gender	Male	60	70	3.75
	Female	26	30	1.71
Age	<1 year	3	3	1.51
	1–4 years	49	57	5.89
	5–11 years	30	35	2.18
	12–15 years	4	5	0.56

### Regional notification rate

**Fig. 3:**
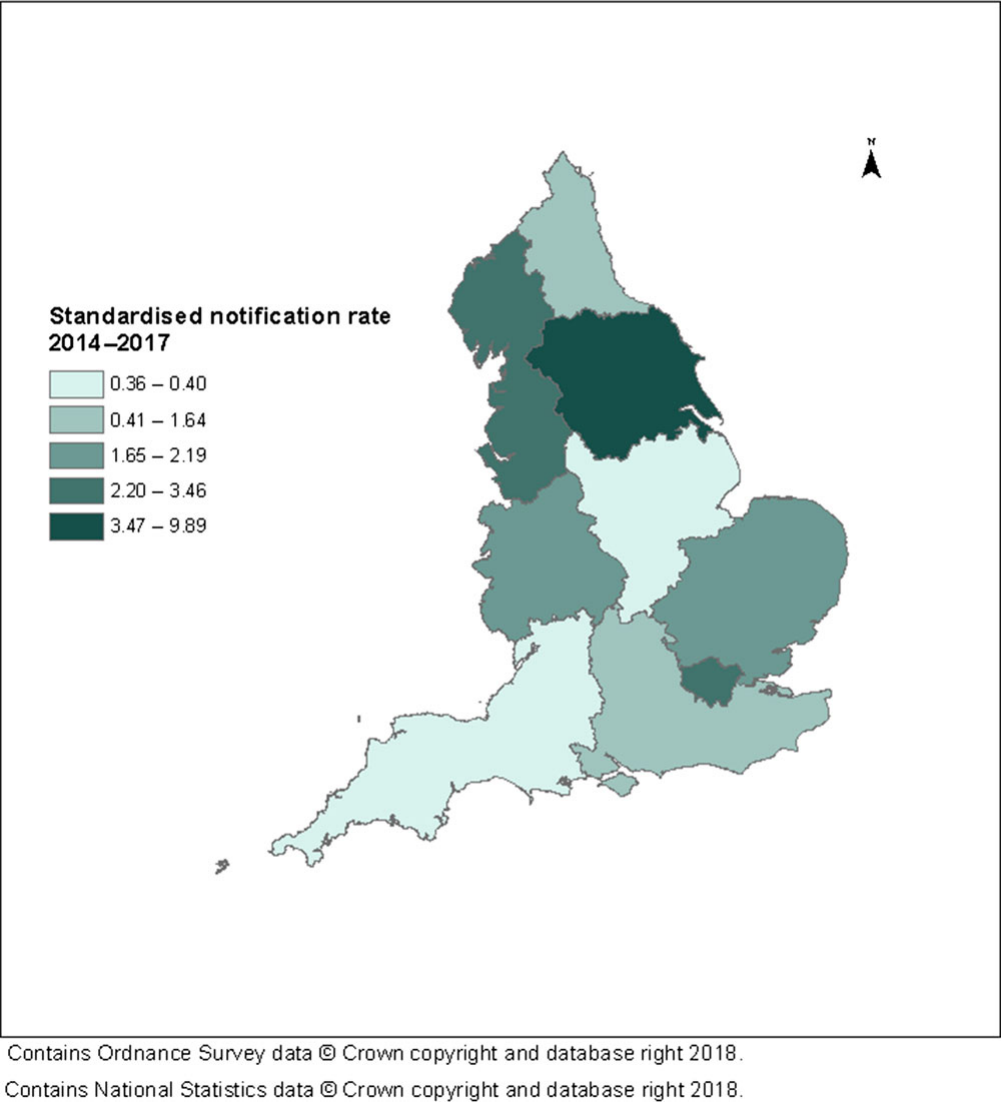
Notification rate of cases (per million 0–15-year-olds) standardised by age and sex, in quintiles, by PHE Centre, England, 1 September 2014–31 August 2017.

Regional 2014–17 crude notification rates ranged from 0.35 per million 0–15-year-olds in the South West to 9.81 per million 0–15-year-olds in Yorkshire and the Humber (see Supplementary Table S1). The DSR was very similar to the crude rate in all regions, ranging from 0.36 to 9.89 per million 0–15-year-olds in the South West and Yorkshire and the Humber, respectively (see Fig. 3). The relative DSR ranged up to 27.47 in Yorkshire and The Humber (see Supplementary Table S1).

### Sensitivity analysis

The notification rate was 3.46 per million in 0–15-year-olds. Distribution of case characteristics and relative risks were almost unchanged (see Supplementary Table S2). The regional distribution of cases was also very similar to the main analysis, except the lowest rate was now observed in the East Midlands (0.39 per million) (see Supplementary Table S3).

## Discussion

### Main finding of this study

Our findings confirm that some children in England still have significant exposure to lead, with 23–37 cases with a BLC ≥0.48 μmol/l (10 μg/dl) reported to PHE LPIC each year, an annual rate of 2.76 per million 0–15-year-olds. Notification rates were higher amongst males aged 1–4 years, and children in deprived areas. Twelve percent of cases were part of a household cluster, and consecutive cases could be older or younger than the index. There was also a 27-fold variation in the rate of notification between regions with the lowest and highest incidence.

### What is already known on this topic

The 22 children reported in the first year of LPIC (September 2014–August 2015) was similar to the 18–19 per year reported for England to the SLiC study (2010–12).^18^ Increased numbers reported to LPIC in years 2–3 most likely reflect familiarity with the reporting mechanism or increased clinician awareness to test for lead exposure, rather than a true increase in lead exposed children. SLiC also reported a similar median age (4 years), and predominance of male gender (62%), but a lower percentage resident in the most deprived quintile (27%, compared with 48%).^18^ Predominance of cases in children by male gender, aged 0–4, and low socio-economic status (SES) has been described using routine hospitalisation data from England,^23^ and survey data from the USA.^6^ Lead exposure correlates with deteriorating and older housing,^11^ and other factors that may also mediate a relationship with lower SES.^11^ An association between greater deprivation and diagnosis with lead exposure therefore seems more likely. The discrepancy with the SLiC study may therefore be due to chance, given the small number of cases. This finding indicates lead exposure may compound existing inequalities. Finally, the median BLC was 1.07μmol/l (21.2 μg/dl) in SLiC, and <1.60 μmol/l in 75% of cases,^18^ broadly similar to our study and consistent with less severe or sub-clinical presentation. Indeed, data from SLiC suggests BLC testing was performed due to the child’s exposure history and behaviours (particularly the presence of pica) in 78%, and/or developmental delay in 28%.^18^ Similarly, pica was the indication for BLC testing in 58% of a sample of LPIC cases in 2014–15.^24^ Paint and soil were the most commonly ingested substances thought to have resulted in exposure.^24^

### What this study adds

We have described epidemiology from a larger number of cases and over a longer period than the SLiC study. Geographic data also allowed us to reveal large differences in notification incidence by region. Explanations for this variation include differences in: distribution of population-level risk factors or lead hazards; likelihood of clinicians to test BLC; and case ascertainment by LPIC. Case numbers do not clearly relate to previous (albeit dated) regional estimates of lead dust concentrations in soil/households,^25^ a strong predictor of paediatric BLCs,^25^ from which we would expect an excess of cases in London rather than Yorkshire and the North West. As noted above, our findings are consistent with awareness of child risk factors (pica/developmental delay) as a cause for, or consequence of lead exposure, driving testing. With no England national guidelines on testing for lead in children presenting with pica or developmental delay, testing practice and policies will be locally determined. The effect can be significant. For example, clinician education and electronic prompts to paediatricians and GPs to consider BLC testing in children with pica instituted by Leeds SAS laboratory (based in Yorkshire and Humber) following the death of a child from lead exposure (the same case as in our results)^26^ resulted in a 90% increase in BLC test requests in the subsequent 12 months.^27^ This may also partly explain the higher case incidence in Yorkshire and Humber. Additional evidence on clinician knowledge about lead exposure, test indication, BLC-testing rates, and the percentage tested with BLC above the action level is needed.

### Limitations of this study

Case ascertainment is likely to significantly under-estimate the real prevalence of lead exposed children, and case characteristics may not be representative of all children with lead exposure in England due to: bias towards children with better-known risk factors for exposure or more severe presentations; the 0.48 μmol/l (10 μg/dl) case definition cut-off introducing selection bias towards children with higher BLCs; the small number of children notified; testing outside of participating SAS laboratories, or non-reporting; and because notification is not a statutory requirement. To accurately estimate prevalence, approaches with fewer biases, such as population surveys, should be considered. If testing outside of participating laboratories or non-reporting differed by region this may partly explain the variation seen between these areas. However, 70% of all cases reported to the SLiC study, which utilised multiple routes of case reporting, were notified by SAS-laboratories, indicating they perform the large majority of BLC testing in children in England.^18^ Results of the sensitivity analysis, including cases not reported by SAS laboratories, did not differ greatly from the main analysis. Together, this suggests PHE was likely notified of the majority of laboratory-confirmed cases, and the characteristics and geographic distribution of cases may not change greatly from ascertainment of the remainder. We used new reports of raised BLCs to define new cases, but it is possible a case may have been diagnosed prior to the system being implemented and then only reported on repeat testing after system initiation, leading to an over-estimate of period incidence. However, the 6-month implementation period means laboratories would have been likely to have already reported such prevalent cases prior to our period of interest. We defined household clusters using case postcode and surname, not address, which may have resulted in misclassification if households with shared surnames were in close proximity. Finally, we did not collect data on some important case characteristics, such as ethnicity, clinical characteristics, testing indication and exposure sources; collation of these data remains a future system objective.

## Conclusions

Our surveillance system identified a small number of children each year with laboratory-confirmed elevated BLCs. Children aged 1–4 years, boys and children from deprived areas may be at higher risk of lead exposure. Together with findings from SLiC and other evidence, this knowledge should inform a wider prevention strategy. Our findings are most relevant to populations likely to experience the worst exposure to lead, such as children with pica or developmental delay. Suitable primary prevention measures suggested for high-risk groups include early housing risk assessment and risk-reduction,^28^ and provision of prevention advice to parents by clinicians.^29^ We observed a heterogeneous regional notification rate, which may be due to, at least in part, varying clinician awareness and suspicion of lead exposure in children presenting with neuro-behavioural problems. Opportunities for secondary prevention include improving clinician awareness of risk factors, exposure sources and clinical presentations of lead exposure. We also recommend clinicians should have a low threshold for screening at-risk children and household contacts of cases. Finally, children in England with lead exposure at or above the action level of 0.48 μmol/l (10 μg/dl) should be notified to PHE for case management and surveillance. These recommendations for clinicians should be made in national clinical guidelines to overcome potential regional practice variations. Limitations of our surveillance methods mean we cannot confidently estimate the risk of lead exposure for the wider under-16 population. Participation of further laboratories, and lowering the action level to <0.48 μmol/l (10 μg/dl) would allow a more comprehensive assessment of lead exposure in children in England. A lower level would follow international precedent to lower action levels for public health case reporting,^30–33^ reflecting the growing evidence base that lead exposure even below <0.48 μmol/l (10 μg/dl) results in toxicity.^12^ PHE are, in conjunction with clinicians and laboratory medicine specialists, currently considering the evidence for this and how it could be best implemented.

## Supplementary Material

fdz024_supp_tables
