# Supplementary material for: Case epidemiology from the first three years of a pilot laboratory-based surveillance system for elevated blood-lead concentrations among children in England, 2014–17: implications for public health action
Source: J Public Health (Oxf). 2019 May 24;42(3):542–9. doi: 10.1093/pubmed/fdz024 (PMC7435212; doi:10.1093/pubmed/fdz024)
Supplement: fdz024_supp_tables [file fdz024_supp_tables.docx]

Supplementary table 1. Counts and crude and standardised average notification rate^†^ of cases (per million 0-15 year old children) by PHE Centre and year of notification, England 01 Sept 2014- 31 Aug 2017

| Region | Cases 2014/15 | Cases 2015/16 | Cases 2016/17 | Cases 2014-17 | Crude notification rate of cases (per million per year) 2015-17 | Age-sex directly standardised rate | Relative DSR** |
| --- | --- | --- | --- | --- | --- | --- | --- |
| South East* | 6 | 0 | 2 | 8 | 1.62 | 1.64 | 4.56 |
| London* | 2 | 6 | 8 | 16 | 3.02 | 2.93 | 8.14 |
| South West | 0 | 0 | 1 | 1 | 0.35 | 0.36 | 1.00 |
| West Midlands* | 2 | 3 | 1 | 6 | 1.78 | 1.79 | 4.97 |
| East Midlands | 1 | 0 | 0 | 1 | 0.39 | 0.40 | 1.11 |
| North West | 2 | 4 | 8 | 14 | 3.45 | 3.46 | 9.61 |
| North East | 0 | 1 | 1 | 2 | 1.43 | 1.42 | 3.94 |
| Yorkshire & Humber* | 8 | 8 | 14 | 30 | 9.81 | 9.89 | 27.47 |
| East of England | 2 | 4 | 2 | 8 | 2.19 | 2.19 | 6.08 |
| ENGLAND | 23 | 26 | 36 | 86 | 2.76 | NA | NA |

**PHE Centres where a SAS laboratory that participates in the surveillance system is situated; † The numerator for this indicator is incident cases in 2014-17, and the denominator is the summed mid-year estimate of the 0-15 population for 2015 multiplied by 3. Cases allocated to PHE Centre according to postcode of residence; DSR = Directly standardised rate; **DSR for this region divided by DSR for South West*

Supplementary Table 2. Count and percentage of cases of cases of exposure to lead, and notification rates by descriptive characteristic, England 1 Sept 2014 – 31 Aug 2017 (n=108)

| Characteristic | Sub-group | Count of cases | Percent | Rate per million |
| --- | --- | --- | --- | --- |
| Index of Multiple Deprivation of residence | 1- Least Deprived  2  3  4  5 – Most deprived | 7  10  15  22  54 | 6  9  14  20  50 | 1.22  1.78  2.58  3.40  7.08 |
| Gender | Male  Female | 76  32 | 70  30 | 4.75  2.10 |
| Age | <1 year  1-4 years  5-11 years  12 years + | 3  62  38  5 | 3  57  35  5 | 1.51  7.46  2.76  0.70 |

Supplementary Table 3. Counts and crude average notification rate^†^ of cases (per million 0-15 year old children) by PHE Centre and year of notification, England 1 Sept 2014- 31 Aug 2017

| Region | Cases 2014/15 | Cases 2015/16 | Cases 2016/17 | Cases 2014-17 | Crude notification rate of cases (per million per year) 2015-17 | Relative notification rate** |
| --- | --- | --- | --- | --- | --- | --- |
| South East* | 6 | 0 | 4 | 10 | 2.03 | 5.21 |
| London* | 4 | 6 | 9 | 19 | 3.59 | 9.21 |
| South West | 2 | 0 | 1 | 3 | 1.04 | 2.67 |
| West Midlands* | 3 | 3 | 1 | 7 | 2.09 | 5.36 |
| East Midlands | 1 | 0 | 0 | 1 | 0.39 | 1.00 |
| North West | 4 | 6 | 10 | 20 | 4.93 | 12.64 |
| North East | 0 | 1 | 1 | 2 | 1.43 | 3.67 |
| Yorkshire & Humber* | 10 | 8 | 18 | 36 | 11.78 | 30.21 |
| East of England | 2 | 5 | 3 | 10 | 2.74 | 7.03 |
| ENGLAND | 32 | 29 | 47 | 108 | 3.46 | NA |

**PHE Centres where a SAS laboratory that participates in the surveillance system is situated; † The numerator for this indicator is incident cases in 2014-17, and the denominator is the summed mid-year estimate of the 0-15 population for 2015 multiplied by 3. Cases allocated to PHE Centre according to postcode of residence;**Crude rate for this region divided by rate for East Midlands*
